# An Unusual Presentation of Extramedullary Relapse Following Blinatumomab in Philadelphia Chromosome-Positive Acute Lymphoblastic Leukemia

**DOI:** 10.7759/cureus.23262

**Published:** 2022-03-17

**Authors:** Sanae Daghri, Mounia Bendari, Nadia Belmoufid, Anass Yahyaoui, Maryame Ahnach

**Affiliations:** 1 Department of Hematology, Faculty of Medicine, Mohammed VI University of Health Sciences (UM6SS) Cheick Khalifa International University Hospital, Casablanca, MAR; 2 Department of Biology, National Reference Laboratory, Mohammed VI University of Health Sciences (UM6SS), Casablanca, MAR

**Keywords:** multiple sites, nipple infiltration, blinatumomab, acute lymphoblastic leukemia (all), extramedullary relapse

## Abstract

In adult patients, extramedullary relapse (EMR) in B-acute lymphoblastic leukemia (B-ALL) has a pejorative prognosis, especially after allogeneic hematopoietic stem cell transplantation (allo-HSCT). Blinatumomab, a bispecific CD3/CD19 antibody, is approved for relapsed/refractory acute lymphoblastic leukemia (ALL) and has proven its efficacy with good complete response (CR) rates and molecular responses in several trials. Unusual sites of relapse following treatment with blinatumomab for ALL are rarely reported. We describe the case of a 23-year-old male with B-ALL characterized as Philadelphia chromosome-positive without extramedullary lesions at diagnosis. He benefited from a matched-related donor allo-HSCT at first remission. A relapse in the bone marrow and central nervous system was diagnosed four months later. A treatment with blinatumomab was initiated with the obtention of CR after one cycle. During the third cycle of blinatumomab, multiple sites of EMR occurred initially with a painless swelling appearing in the areolas and the nipples, followed by bilateral testicular hypertrophy and moderate paraplegia. A diagnosis of leukemic infiltration on the areola-nipple complex was made by cytological analysis of the fine-needle aspiration of the left areola. The analysis of bone marrow was normal, but molecular BCR-ABL was positive. Systemic chemotherapy with hyper-CVAD (cyclophosphamide, vincristine, doxorubicin, and dexamethasone) and cycles of blinatumomab with nilotinib was initiated in association with intrathecal chemotherapy and whole-brain radiation therapy. Clinical, molecular, and central nervous remissions were obtained.

We report this case to describe multiple sites of EMR of B-ALL with atypical breast infiltration in an adult male patient following treatment with blinatumomab.

## Introduction

The efficacy of treatment in patients with newly diagnosed B-acute lymphoblastic leukemia (B-ALL) has improved significantly in recent decades. This has resulted from better knowledge of the disease severity‘s factors, the use of multi-agent chemotherapy protocols, and recourse to allogeneic hematopoietic stem cell transplantation (allo-HSCT) for eligible patients [1]. However, despite this progress, patients who relapse or are refractory to initial treatments still have a relatively poor prognosis with relapse rates ranging between 30% and 70% following allo-HSCT [2,3]. The development of new promising novel therapeutics, based on immunotherapy, has improved these results. Among this treatment, blinatumomab is a bispecific antibody that binds CD19 on the surface of B-cells and CD3 on the surface of T-cells, thereby leading to the lysis of the targeted B cells achieving a complete response (CR) rate up to 69% in relapsed/refractory (R/R) B-ALL [4-7]. Unfortunately, relapse still occurs following an initial response in approximately 50% of patients, either as an isolated relapse or with bone marrow involvement. Extramedullary relapse (EMR) following blinatumomab is less characterized, and the most frequent sites of EMR such as the central nervous system (CNS) and testis are referred to as acute lymphoblastic leukemia (ALL)-blast sanctuaries. Rarely, unusual relapse sites following blinatumomab have been reported, including the lungs, kidneys, and spleen [8-10]. However, extramedullary leukemic infiltration of the breast or in the nipple-areolas complex, especially in an adult male patient, at diagnosis or relapse is extremely rare. Herein, we report a case of multiple sites of extramedullary localization with atypical breast infiltration at relapse of B-ALL in an adult male patient following treatment with blinatumomab.

## Case presentation

A 23-year-old male patient with no family history and no comorbidities was diagnosed with B-ALL by bone marrow biopsy and flow cytometry. Cytogenetic analysis performed on bone marrow aspirate by conventional karyotyping was positive for BCR/ABL translocation, and FISH detected t(9;22) (BCR-ABL1). He received combination chemotherapy following GRAAPH protocol including imatinib 100 mg/day and CNS prophylaxis. A cytological and molecular complete remission (mCR) was achieved. He successfully underwent a matched-related donor allo-HSCT with non-total body irradiation (TBI) based conditioning regimen.

**Figure 1:**
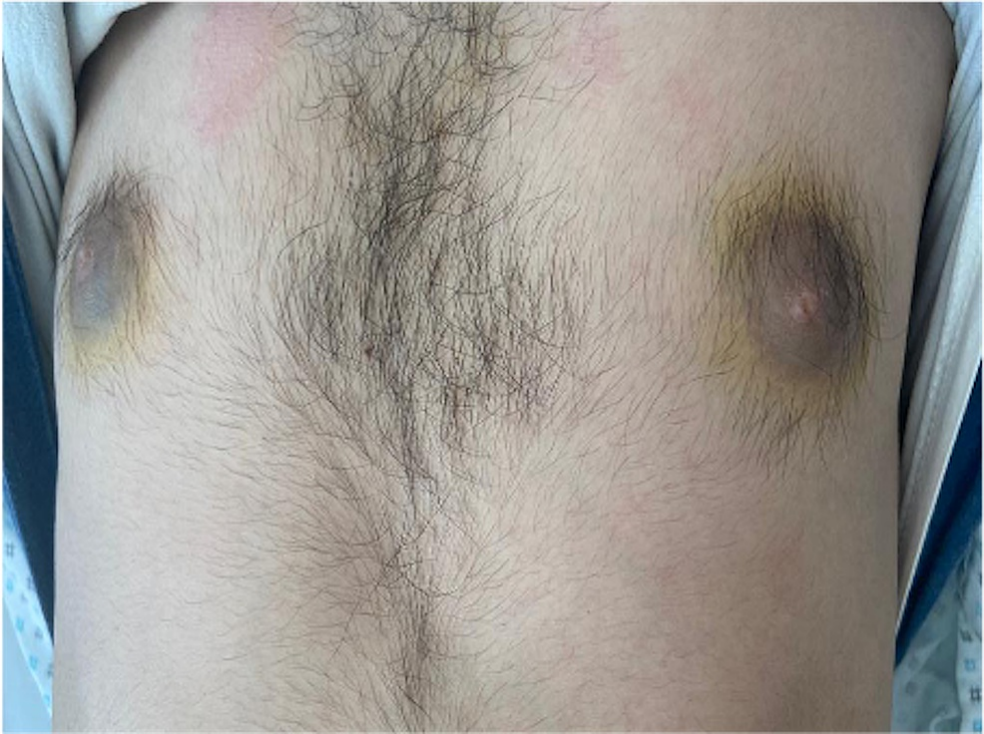
Induration involving the two nipple-areola complex

**Figure 2:**
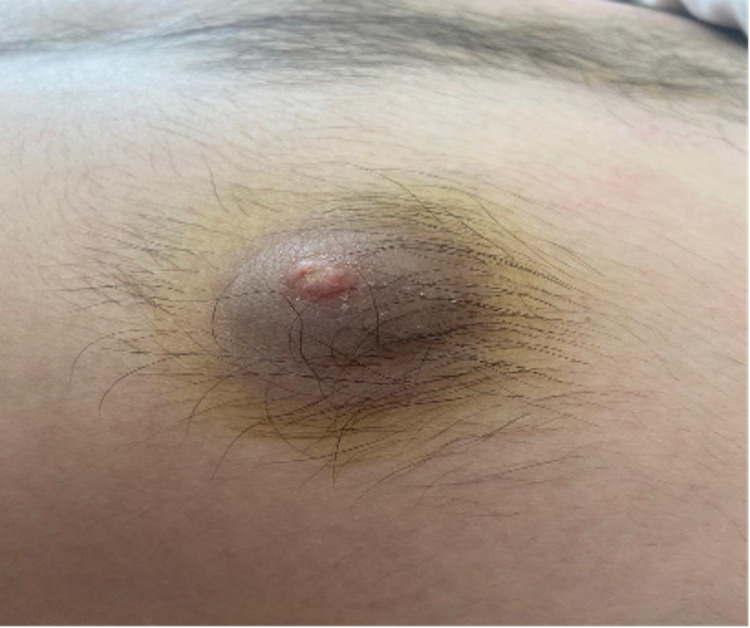
Swelling involving the left nipple-areola

**Figure 3:**
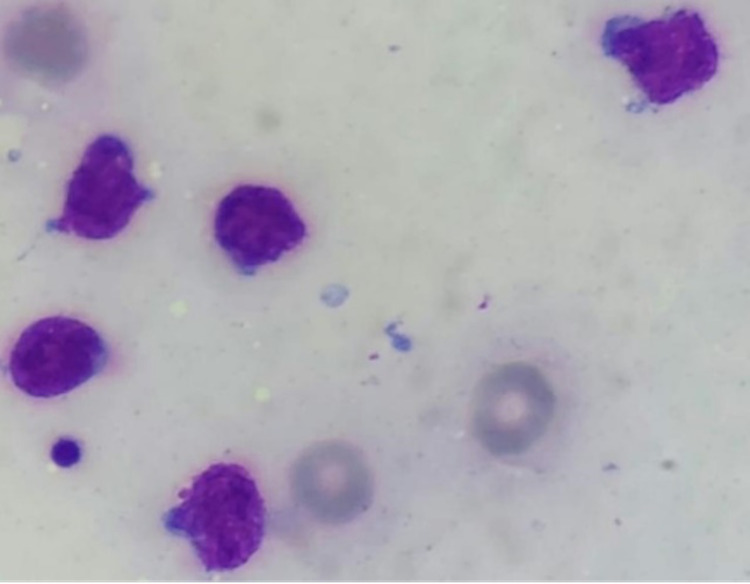
Lymphoblasts with large nuclei, fine chromatin, and minimal cytoplasm in cytological analysis of the needle aspiration

Four months later, the first relapse occurred with fatigue, epistaxis, facial paralysis, and pancytopenia. The bone marrow aspiration confirmed the relapse of B-ALL with cytogenetic detecting t (9,22) and detection of BCR-ABL in molecular analysis. The lumbar puncture revealed the presence of blasts in the cerebrospinal fluid (CSF) confirming the relapse in CNS. He was treated with blinatumomab by continuous infusion (9ug per day for 7 days and then 28ug per day for 21 days) administered for four weeks followed by a two-week treatment-free interval associated with nilotinib and intrathecal chemotherapy. No cytokine release syndrome or neurologic adverse events were reported. After one cycle, he achieved complete remission in the bone marrow and CSF associated with cytogenetic and molecular remission. While receiving the third cycle of blinatumomab, he initially developed an increased swelling on the two nipple-areola complexes followed by headaches and motor and sensory impairment of the lower limbs. On physical examination, the two breasts were symmetrical with a painless swelling firm in consistency in the areolas and the nipples (Figures 1, 2), with bilateral testicular hypertrophy and moderate paraplegia. The fine needle aspiration of the left areola revealed infiltrating blast cells (Figure 3).

**Figure 4:**
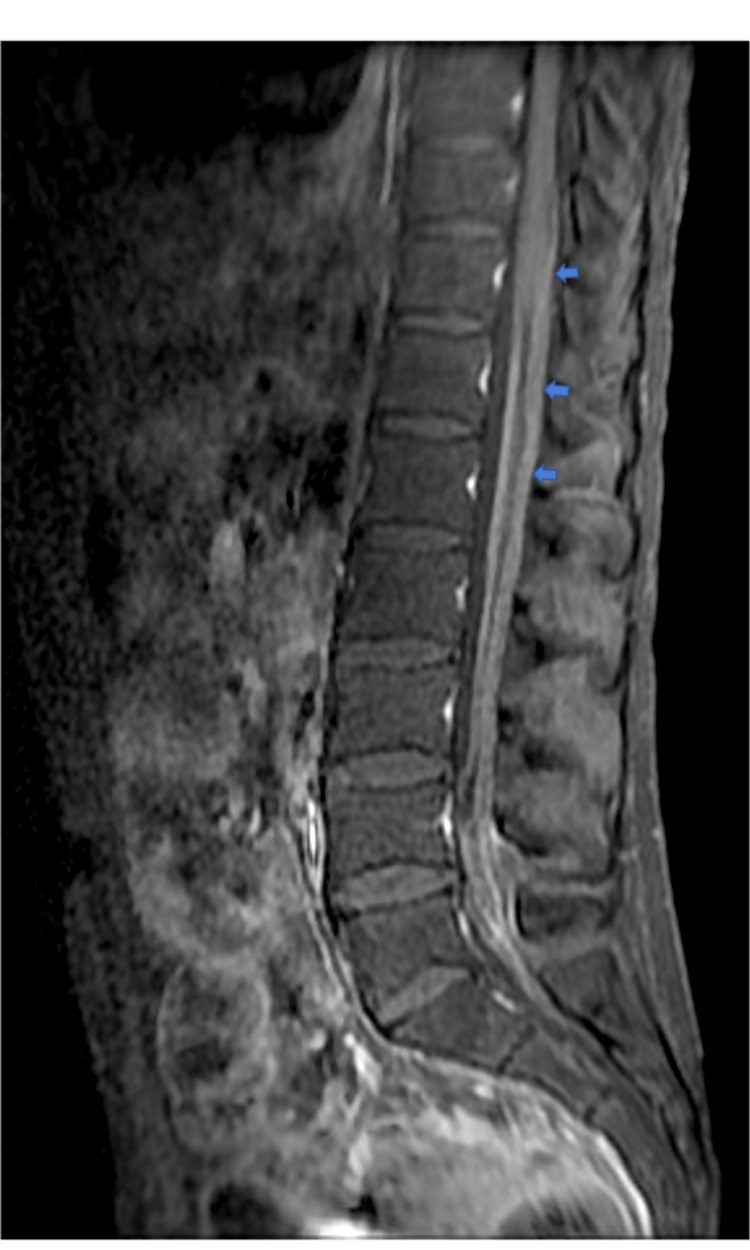
Post-contrast T1-weighted MRI image in sagittal plane demonstrating diffuse leptomeningeal enhancement (shown by arrows) at the time of presentation

A testicular ultrasound revealed a hypoechogenic nodule suspected of malignant infiltration. Post-contrast T1-weighted Magnetic resonance imaging (MRI) image in sagittal plane confirmed a diffuse leptomeningeal enhancement (Figure 4).

The analysis of bone marrow was normal, but molecular BCR-ABL was positive.

**Figure 5:**
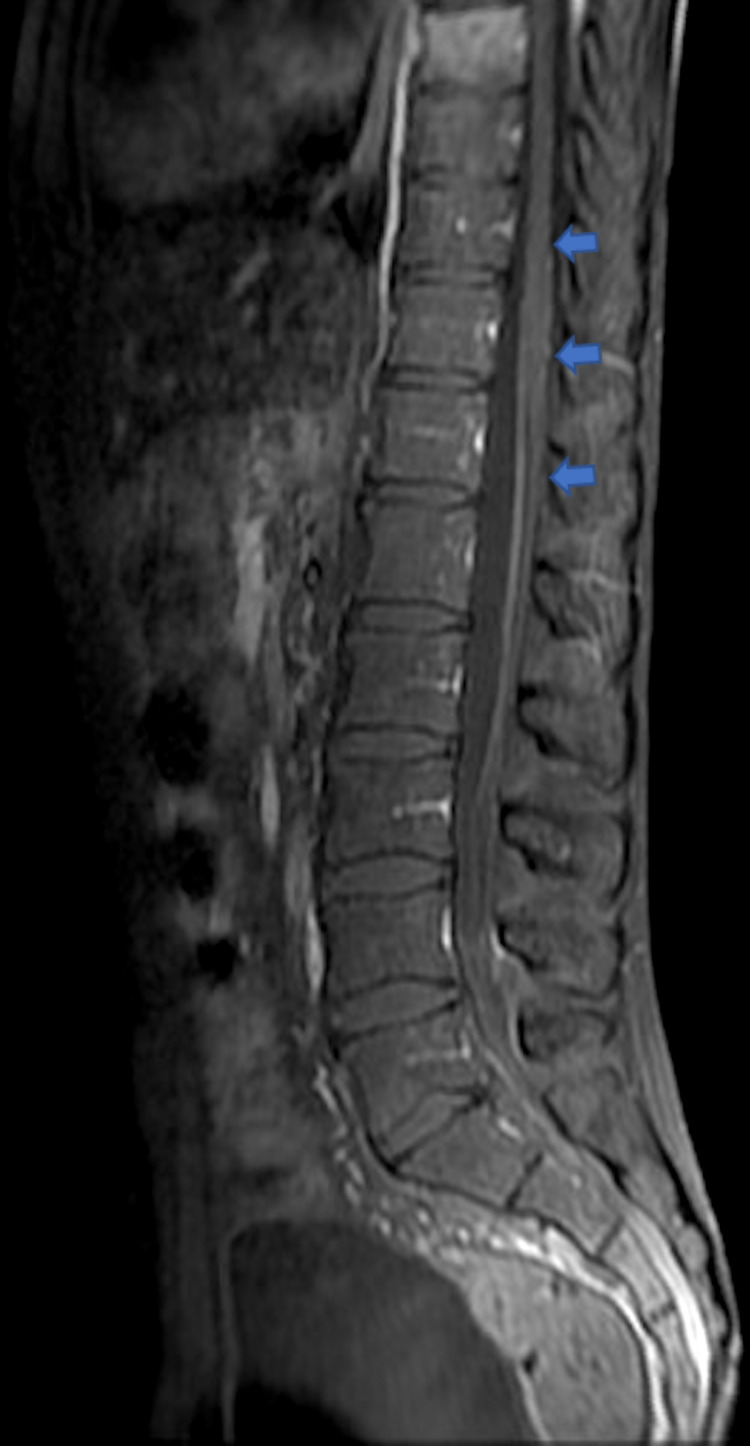
Post-contrast T1-weighted MRI image in sagittal plane demonstrating regression of the leptomeningeal infiltration (shown by arrows) after treatment

Considering the EMRs, hyper-CVAD (cyclophosphamide, vincristine, doxorubicin, and dexamethasone)/blinatumomab regimen with nilotinib was initiated in association with intrathecal chemotherapy and whole-brain radiation therapy. After four cycles, the evolution was characterized by the obtention of complete regression of testicular hypertrophy and swelling of the two nipples, along with a radiological neurological remission (Figure 5) associated with complete molecular remission.

The treatment maintenance was continued with blinatumomab and nilotinib.

## Discussion

We reported a case of multiple sites of EMR in an adult male patient following treatment with blinatumomab for B-ALL. The most common sites reported for EMR in ALL are the central nervous and testis. In our patient, relapse occurred after three cycles of blinatumomab in the breast, testis, and CNS.

Several studies have previously reported and described EMR after treatment with blinatumomab. Notably, Aldoss et al. [9,11] in two retrospective studies including patients treated with blinatumomab for R/R ALL reported EMR rates ranging from 32% to 49% among initial responders to blinatumomab. Sites of EMR included the CNS, kidney, lymph node, muscle, chest wall, and nasopharynx. In another recent study by Lau et al. including adult patients with R/R B-ALL or with minimal residual disease positive and treated with blinatumomab, the EMR rate was 20% and included lymph nodes, pancreas, adrenal gland, kidneys, liver, left parotid gland, and brain [12]. In our case, the patient presented multiple EMR sites. To our knowledge, breast involvement in B-ALL has never yet been described in a male adult patient. However, in the pediatric population, breast involvement of B-ALL at diagnosis or relapse has been described in only a few cases [13].

The mechanism of these EMRs post-blinatumomab remains poorly understood but could be explained by several hypotheses. The first hypothesis is a pharmacokinetic mechanism with a decrease in the concentration of blinatumomab in some extramedullary sites. The second is the inability of blinatumomab to penetrate some tissues, and at last an immune phenomenon with a lack of the recruitment of T cells by blinatumomab to non-hematopoietic tissues [10]. The risk factors for EMR after blinatumomab remains poorly identified. However, a history of extramedullary involvement, phenotype T-cell acute leukemia, and a non-TBI conditioning regimen proved to be a significant risk factor for EMR in patients after allo-HSCT [14]. Nevertheless, in our patient, retreatment with blinatumomab associated with systemic chemotherapy was effective in regression of the swelling in the nipples combined with a neurological response and the obtention of molecular remission. In concordance with studies reporting the efficacy of reintroduction of blinatumomab in patients with R/R B-ALL, a remission rate of 36% suggests that blinatumomab retreatment can be used for patients who have responded to blinatumomab previously [15]. Extramedullary involvement or relapse in ALL remains an unfavorable prognosis; however, chimeric antigen receptor (CAR) T-cell therapy is a possible therapeutic option that could potentially salvage patients experiencing relapse after blinatumomab therapy [16].

This is a rare case of extramedullary leukemic infiltration of the breast in an adult male patient as the first manifestation of multiple sites of relapsed B-ALL after treatment with blinatumomab. In the literature, breast involvement of B-ALL has been described only in the pediatric population. Identifying specific factors and mechanisms of EMR is required to propose an adequate therapeutic approach.

## Conclusions

A report on this rare case aims to highlight the uncommon infiltration in the nipples as the first manifestation of multiple sites of relapsed B-ALL following treatment with blinatumomab. Through our case, we illustrate the particularity of this particular site of EMR. Uncommon EMR, as in our patient, may hinder early diagnosis. Clinicians should therefore consider this for early detection and may add it to other unusual extramedullary sites of relapse. Moreover, further studies are needed to identify the potential sites of EMR after blinatumomab, its risk factors, and prognostic indicators that may be helpful in the early diagnosis of relapse and planning for therapy.
